# NPAS4 Exacerbates Pyroptosis via Transcriptionally Regulating NLRP6 in the Acute Phase of Intracerebral Hemorrhage in Mice

**DOI:** 10.3390/ijms24098320

**Published:** 2023-05-05

**Authors:** Dan Jian, Le Qin, Hui Gan, Shuyue Zheng, Han Xiao, Yuhao Duan, Mi Zhang, Ping Liang, Jing Zhao, Xuan Zhai

**Affiliations:** 1 Department of Neurosurgery, National Clinical Research Center for Child Health and Disorders, Ministry of Education Key Laboratory of Child Development and Disorders, Chongqing Key Laboratory of Pediatrics, Children’s Hospital of Chongqing Medical University, Chongqing 400010, China; jd1101350878@163.com (D.J.); qinle_qinyue@163.com (L.Q.); ganhui@cqmu.edu.cn (H.G.);; 2 Center for Neuroscience Research, School of Basic Medicine, Chongqing Medical University, Chongqing 400016, China

**Keywords:** intracerebral hemorrhage, pyroptosis, NPAS4, NLRP6 inflammasome

## Abstract

Intracerebral hemorrhage (ICH) is a severe cerebrovascular disease with a high disability rate and high mortality, and pyroptosis is a type of programmed cell death in the acute phase of ICH. Neuronal Per-Arnt-Sim domain protein 4 (Npas4) is a specific transcription factor highly expressed in the nervous system, yet the role of NPAS4 in ICH-induced pyroptosis is not fully understood. NLR family Pyrin-domain-containing 6 (NLRP6), a new member of the Nod-like receptor family, aggravates pyroptosis via activating cysteine protease-1 (Caspase-1) and Caspase-11. In this study, we found that NPAS4 was upregulated in human and mouse peri-hematoma brain tissues and peaked at approximately 24 h after ICH modeling. Additionally, NPAS4 knockdown improved neurologic dysfunction and brain damage induced by ICH in mice after 24 h. Meanwhile, inhibiting NPAS4 expression reduced the levels of myeloperoxidase (MPO)-positive cells and Caspase-1/TUNEL-double-positive cells and decreased cleaved Caspase-1, cleaved Caspase-11, and N-terminal GSDMD levels. Consistently, NPAS4 overexpression reversed the above alternations after ICH in the mice. Moreover, NPAS4 could interact with the *Nlrp6* promoter region (−400–−391 bp and −33–−24 bp) and activate the transcription of *Nlrp6*. Altogether, our study demonstrated that NPAS4, as a transcription factor, can exacerbate pyroptosis and transcriptionally activate NLRP6 in the acute phase of intracerebral hemorrhage in mice.

## 1. Introduction

Intracerebral hemorrhage (ICH) is a severe cerebrovascular disease with a poor prognosis, a high disability rate, and high mortality, resulting in a heavy burden on patients’ families and society [1,2]. The poor prognosis of ICH is mainly attributed to a primary brain injury caused by the spatial occupation of the hematoma and a secondary brain injury (SBI) triggered by the toxic stimulation of hematoma components [3,4]. Additionally, two types of brain injuries are involved in nerve cell death and eventually lead to deficits in neurological function [5]. Numerous studies have investigated the effectiveness of the surgical removal of hematomas; however, such effectiveness involves many factors and is not very definitive [6]. At the same time, there are still few effective medical therapies for alleviating an SBI [7]. Therefore, the exploration of endogenous proteins related to neuronal death may contribute to basic research on the pathogenesis of intracerebral hemorrhage and provide new targets for clinical treatments.

Various forms of cell death occurring after intracerebral hemorrhage have been identified, including apoptosis, necrosis, and ferroptosis in humans and experimental animals [8,9,10,11]. Pyroptosis is a novel form of inflammasome-mediated programmed cell death [12]. The classic pyroptosis pathway principally depends on cysteinyl-aspartate-specific proteinase 1 (Caspase-1) [13]. Briefly, when cells are subjected to harmful stimuli, inflammasomes composed of NLRs (NOD-like receptors), apoptosis-associated speck-like protein (ASC), and Caspase-1 are activated, leading to the shear-induced activation of Caspase-1 [14,15]. Activated Caspase-1 not only participates in the innate immune response and mediates the shear and release of inflammatory factors but is also involved in the activation of pyroptosis executive protein gasdermin D (GSDMD), which, subsequently, induces cell membrane perforation and pyroptosis [16,17].

NLR family pyrin domain protein 6 (NOD-like receptor pyrin-domain-containing 6, NLRP6) is a new member of the human NLR protein family [18,19]. Like other NLR family members, NLRP6 can form inflammasome and regulate the activation of Caspase-1 in the body’s immune response [20]. More interestingly, NLRP6 can also recruit Caspase-11, further promoting the activation of Caspase-1 [21,22]. Our previous study found that inhibiting NLRP6’s expression level could alleviate brain injury induced by ICH, suggesting that the activation of the NLRP6 inflammasome aggravates brain damage after intracerebral hemorrhage [23].

Neuronal Per-Arnt-Sim domain protein 4 (NPAS4), a member of the basic helix–loop–helix (BHLH)-PAS protein family, is a specific transcription factor highly expressed in the nervous system [24,25]. BHLH-PAS proteins are members of the BHLH transcription factor superfamily that generally contain two structurally conserved PAS domains [26]. Studies have shown that BHLH-PAS proteins participate in many biological processes, such as the development of the central nervous system and the induction of pathological responses after hypoxia [27,28]. More and more studies have revealed that NPAS4 plays an important role in neurological diseases such as ischemic stroke, autism, depression, cognitive disorders, etc. [29,30]. However, the role of NPAS4 in the acute phase of intracerebral hemorrhage in mice still remains unknown. Moreover, we found that there are three theoretical NPAS4-binding sites in the *Nlrp6* promotor region using bioinformatics prediction approach. Hence, we hypothesized that NPAS4 may modulate pyroptosis by regulating the transcriptional expression of *Nlrp6* and the activation of the NLRP6 inflammasome after ICH. In summary, this study investigated the role and potential mechanism of NPAS4 in a brain injury after intracerebral hemorrhage in mice.

## 2. Results

### 2.1. The Spatiotemporal Expression of NPAS4 after ICH

**Figure 1:**
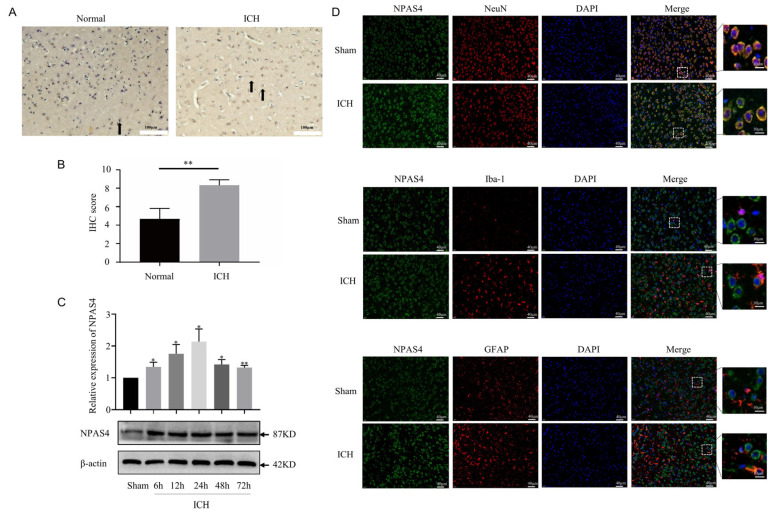
The spatiotemporal expression of NPAS4 after ICH. (**A**,**B**) The expression of NPAS4 around the hematoma of patients with ICH (scale bar = 100 μm, arrow: NPAS4-positive cells); (**C**) protein level of NPAS4 in the peri-hematoma tissues of ICH mouse model detected via Western blotting at 6, 12, 24, 48, and 72 h compared with sham group; (**D**) the cellular localization of NPAS4 in the perihematomal region at 24 h after ICH using double immunofluorescent staining (scale bar = 40 μm or 10 μm). * *p* < 0.05 vs. sham; ** *p* < 0.01 vs. sham or normal.

Representative pictures of immunohistochemical staining (Figure 1A,B) showed that the expression of NPAS4 was significantly increased around the hematoma tissues of patients with ICH compared with that in controls (*p* < 0.01). To investigate the spatiotemporal expression of NPAS4 in the peri-hematoma tissue, Western blotting was performed to detect the protein levels of NPAS4 at 6, 12, 24, 48, and 72 h after ICH compared with a sham control. The results indicated that the NPAS4 expression level started increasing at 6 h and reached a peak at approximately 24 h after ICH (Figure 1C). Therefore, the particular point in time of 24 h after modeling was adopted for follow-up experiments. Double immunofluorescent staining was conducted to reveal the co-localization of NPAS4 protein with NeuN, Iba-1, and GFAP in the brain tissues of the ICH and sham groups. The resulting representative photographs (Figure 1D) showed that ring-shaped NPAS4-positive staining cells mainly co-localized with NeuN-positive cells, whereas co-localization between Npas4 and Iba-1 or GFAP was scarcely observed in the images, which suggested that the NPAS4 protein is primarily expressed in neurons. Taken together, these results revealed that expression of NPAS4, which is predominantly expressed in mouse neurons, was upregulated in the perihematomal brain tissues from both ICH model mice and patients with ICH.

### 2.2. NAPS4 Deficiency Attenuated Neurological Function Deficits, Morphologic Damage, and Neutrophil Infiltration after ICH in Mice

**Figure 2:**
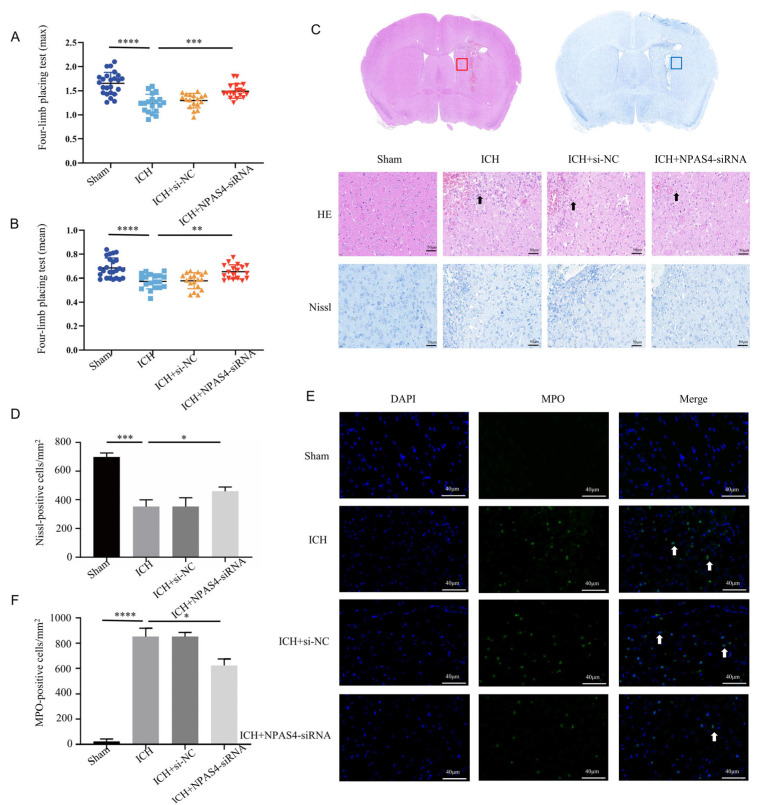
Effects of interfering NPAS4 on neurological outcome, morphologic damage, and neutrophil infiltration at 24 h after ICH modeling. (**A**,**B**) Grip strength tests were conducted to evaluate neurological function (Sham, *n* = 24; ICH, *n* = 19; ICH + NC, *n* = 18; ICH + NPAS4-siRNA, *n* = 18); (**C**,**D**) H&E and Nissl staining were performed to detect brain injuries around hematoma (scale bar = 50 μm; arrows, leukocyte; *n* = 4/group); (**E**,**F**) immunofluorescence staining was used to assess neutrophil infiltration in perihematomal brain areas (scale bar = 40 μm; arrow: MPO-positive cells; *n* = 4/group). Sham—mice without any treatment, ICH—mice without injection before ICH induction; ICH + NC—mice with scrambled siRNA injection at 24 h before ICH induction; ICH + NPAS4-siRNA—mice with NPAS4-siRNA injection at 24 h before ICH induction. The data are shown as mean ± SD. * *p* < 0.05 vs. ICH, ** *p* < 0.01 vs. ICH or sham, *** *p* < 0.001 vs. ICH, and **** *p* < 0.0001 vs. sham group.

Firstly, we injected NPAS4-siRNA fragments (si-503, si-848, and si-2253) into the right lateral ventricles of wild-type mice; then, we screened out the optimum interference fragments (NPAS4-siRNA 848; Figure S1), which could significantly reduce the expression of NPAS4 in mice. To evaluate the effects of NPAS4 deficiency on ICH-induced brain damage, mice were assigned randomly to four groups (sham, ICH, ICH + NC, and ICH + NPAS4-siRNA) and their grip strength values (mean values and max values) were measured in triplicate using a grip strength meter at 24 h after the onset of ICH. Subsequently, the mice were sacrificed, and their brain sections were subjected to hematoxylin and eosin (H&E) and Nissl staining. The grip test (Figure 2A,B and Figure S2) showed that the ICH mice (max values—1.24 ± 0.19 N; mean values—0.57 ± 0.06 N) had more severe muscle weakness than the sham group (max values—1.65 ± 0.23 N; mean values—0.67 ± 0.08 N), and a considerable improvement in the muscle strength of the ICH + NPAS4-siRNA mice (max values—1.49 ± 0.19 N; mean values—0.65 ± 0.06 N) was observed when compared to the ICH model group. Moreover, no significant differences were found between the ICH and ICH + NC groups (max values—1.30 ± 0.15 N; mean values—0.58 ± 0.07 N). The results implied that NPAS4 deficiency could greatly improve neurological function in the ICH model mice. H&E staining (Figure 2C) revealed that the peri-hematoma brain tissues of the ICH and ICH + NC groups were infiltrated by erythrocytes, appeared to be loose, and exhibited karyopyknosis and severe brain edema in contrast to the sham control. In addition, these areas were infiltrated by leukocytes, which can be stained purple to blue and typically have a prominent nucleus and a relatively small size compared to other cells such as neurons and glia. Consistently, Nissl staining (Figure 2C,D) showed that there were fewer and irregularly arranged Nissl-positive neurons in the ICH and ICH + NC model groups. Of note, all these alterations were obviously improved in the ICH + NPAS4-siRNA group, which illustrated that NAPS4 deficiency could effectively alleviate brain injuries in the ICH-mice. Immunofluorescence staining (Figure 2E,F) was performed to assess the degree of neutrophil infiltration by counting the number of MPO-positive cells in the perihematomal brain region. As the representative images show, the infiltration of MPO-positive cells had occurred in both the ICH and ICH + NC groups, whereas these positive neutrophils were hardly seen in the sham group. In addition, there were no significant differences between the ICH and ICH + NC groups. Moreover, fewer MPO-positive cells were found around the hematoma in the ICH + NPAS4-siRNA group than in the ICH group (*p* < 0.05), which revealed that NAPS4 deficiency could reduce the infiltration degree of neutrophils. Altogether, these results indicated that the interference of NPAS4 expression attenuated brain injuries, neurological function damage, and neutrophil infiltration in the ICH model mice, which suggests that NPAS4 played a pernicious role in the ICH model mice.

### 2.3. NAPS4 Deficiency Lessened Pyroptosis after ICH Modeling

**Figure 3:**
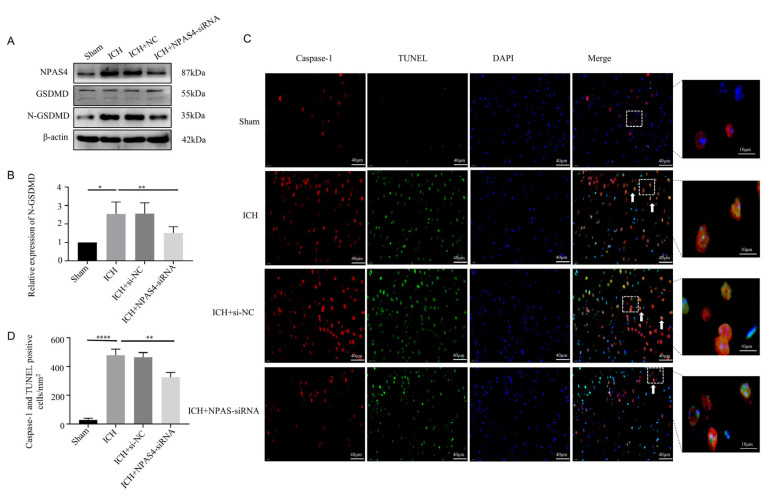
Effect of inhibiting NPAS4 on pyroptosis at 24 h after ICH. (**A**,**B**) Protein level of N-GSDMD was detected via Western blotting and normalized to β-actin; (**C**,**D**) Caspase-1/TUNEL-double-positive cells were evaluated using immunofluorescence staining and TUNEL assay (scale bar = 40 μm or 10 μm; arrows, Caspase-1/TUNEL double positive cell). * *p* < 0.05 vs. sham, ** *p* < 0.01 vs. ICH, and **** *p* < 0.0001 vs. sham.

To specifically explore the function of NPAS4 toward ICH, Western blotting, immunofluorescence staining, and TUNEL assays were performed to investigate the effect of inhibiting NPAS4 on ICH-induced pyroptosis. Western-blotting analysis (Figure 3A,B) revealed that the protein level of N-GSDMD, a pyroptosis executor molecule, was upregulated after ICH modeling when compared to the sham mice (*p* < 0.05), but the treatment of NAPS4-siRNA injection before ICH presented a distinctly reversible alternation in comparison with simply ICH modeling (*p* < 0.01). Besides, the protein levels of GSDMD-FL, pro-Caspase-1 and pro-Caspase-11 were showed none significant differences among these groups (Supplementary Figure S3A–C; *p* > 0.05). Immunofluorescence staining together with TUNEL assays were conducted to further explore the impact of NAPS4 on pyroptosis. The representative pictures (Figure 3C,D) exhibited that the number of Caspase-1/TUNEL-double-positive cells was greatly increased in the ICH group compared with the sham group (*p* < 0.0001). Consistently, the numbers of these counted positive cells were decreased in the ICH + NPAS4-siRNA group in contrast with the ICH group (*p* < 0.01). All these data reveal that inhibiting NAPS4 expression might offer a protective function against ICH mice via attenuating cell pyroptosis.

### 2.4. The Interaction of NPAS4 and Nlrp6 Promoted Inflammasome Activation after ICH

#### 2.4.1. NPAS4 Deficiency Inhibited the Activation of NLRP6 Inflammasome after ICH

**Figure 4:**
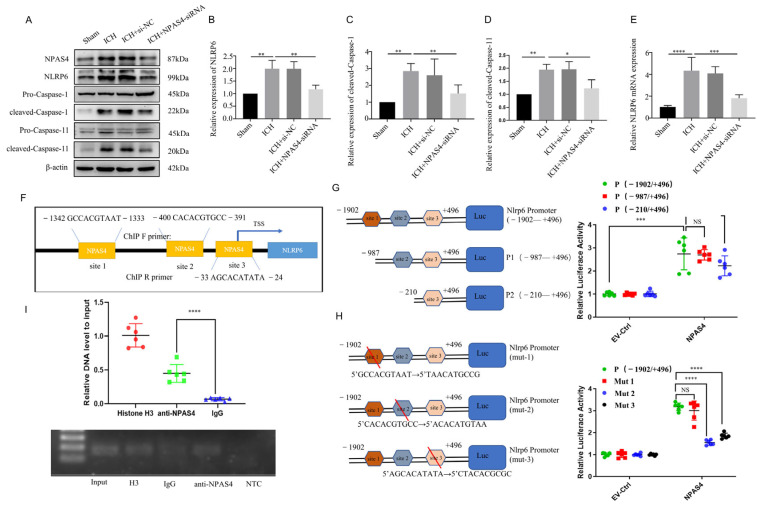
The interaction of NPAS4 and Nlrp6 promoted inflammasome activation after ICH. (**A**–**D**) Western blotting was used to detect the protein levels of NLRP6, cleaved Caspase-1, and cleaved Caspase-11 (*n* = 4/group); (**E**) PCR was performed to detect the expression level of Nlrp6 mRNA; (**F**) the predicted binding sites of transcriptional factor NPAS4 to *Nlrp6* promotor sequence; (**G**,**H**) dual luciferase reporter assay was carried out to explore the interaction of NPAS4 and Nlrp6 (*n* = 6/group) (TSS: transcription start site; site 1: −1342–−1333 bp, site 2: −400–−391 bp, site 3: −33–−24 bp); (**I**) Ch-IP assay in N2A cells was conducted to confirm the interaction of transcription factor NPAS4 with *Nlrp6* promotor (*n* = 6/group) (Input, internal control; H3, positive control; IgG, negative control; NTC, No Template Control). The data are shown as means ± SD. * *p* < 0.05, ** *p* < 0.01, *** *p* < 0.001, and **** *p* < 0.0001. ns, no significant.

In our previous studies, we found that NLRP6 could aggravate brain injuries in an ICH-induced rat model and exaggerate cell pyroptosis in an oxygen glucose deprivation (OGD) model. To explore the regulation of NPAS4 on NLRP6 in vivo, quantitative real-time PCR was conducted to detect *Nlrp6* transcription levels, and Western blotting was carried out to determine the protein levels of NLRP6, cleaved-Caspase-1, and cleaved-Caspase-11 in each group. As shown in Figure 4E, the mRNA expression level of *Nlrp6* was greatly upregulated in the ICH and ICH + NC groups compared with the sham group (*p* < 0.0001), whereas no differences were found between the ICH and ICH + NC groups. However, this alternation was remarkably reversed after NPAS4-siRNA injection before modeling in contrast with the ICH group (*p* < 0.001), which revealed that NPAS4 is a positive regulator of the Nlrp6 gene. As presented in Figure 4A–D, the protein levels of NPAS4, NLRP6, cleaved-Caspase-1, and cleaved-Caspase-11 were significantly increased in the ICH and ICH + NC groups when compared to the sham group (*p* < 0.01), while the treatment of NPAS4-siRNA greatly decreased their expression in comparison with the ICH group (*p* < 0.01). Briefly, these results support the notion that inhibiting NPAS4 downregulates the expression of NLRP6, cleaved-Caspase-1, and cleaved-Caspase-11, indicating that NPAS4 deficiency is likely to attenuate inflammasome activation via regulating NLRP6 expression after ICH.

#### 2.4.2. The Interaction of NPAS4 and Nlrp6 Promotor

To determine how NPAS4 regulates the expression of NLRP6, we predicted and analyzed these cis-acting elements interacted with the transcriptional factor NPAS4 using the Jasper2020 online tool and found that there were three possible NPAS4-binding sites (Figure 4F) in the areas around the *Nlrp6* promotor sequence. Therefore, a dual luciferase reporter assay was carried out to preliminarily explore the interaction of NPAS4 and the *Nlrp6* promotor. Firstly, N2A cells from murine neuroblastoma were divided into two groups and then co-transfected with pGL4.10-*Nlrp6* promotor(fLuc) and pGL4.74(rLuc), respectively, together with NPAS4 overexpression plasmids (pIRES2-*Npas4*-Flag) or empty vector plasmids (EV-Ctrl). Forty-eight hours later, firefly and renilla luciferase activity was detected and analyzed to estimate the *Nlrp6* transcription level. As presented in Figure 4G, relative luciferase activity (firefly/renilla activity) was considerably increased in the plasmids (pIRES2-*Npas4*-Flag) compared with the empty vector (*p* < 0.001), which demonstrated that the NPAS4 protein upregulated the transcriptional activity of the *Nlrp6* promotor (−1902–+496 bp). Secondly, N-terminally truncated *Nlrp6* promotor sequences (P1: −987–+496 bp; P2: −210–+496 bp) were constructed to investigate the possible NPAS4 action sites according to a schematic diagram (Figure 4G). Subsequently, N2A cells (pIRES2-*Npas4*-Flag or empty plasmids, rLuc) were transfected with plasmids (fLuc) containing an intact promotor sequence (−1902–+496 bp) or two designed truncations; then, luciferase activity in each group was tested using dual luciferase reporter assay kits. From Figure 4G, we found that both *Nlrp6* promotor truncations showed no obvious alternations in relative luciferase activity when compared to the intact *Nlrp6* promotors (*p* > 0.05). Moreover, there was no significant difference between P1 and P2 truncations (*p* > 0.05). The results indicated that NPAS4 protein might interact with any one of the three binding sites predicted above in the *Nlrp6* promotor. To further explore the specific NPAS4-*Nlrp6* interaction sites, *Nlrp6* promotor mutants (site1→mut1; site2→mut2; site3→mut3) were constructed by respectively mutating ten bases of the predicted binding-sites (Figure 4H). Then, N2A cells (pIRES2-*Npas4*-Flag or empty plasmids, rLuc) were co-transfected with either pGL4.10-*Nlrp6* promotor mutants (fLuc; mut1 or mut2 or mut3) or the original control vector, and luciferase activity in every group was detected to evaluate the effect of NPAS4 on Nlrp6 transcriptional activation level. The results (Figure 4H) showed that in the NPAS4 overexpression group, the relative luciferase activity of mutant2 and mutant3 was significantly decreased (*p* < 0.0001), while no difference was observed in mut1 (*p* > 0.05) compared with the original control. Consequently, it was surmised that NPAS4 is more likely to activate the transcription of *Nlrp6* through interacting with site2 (−400–−391 bp: CACACGTGCC) and site3 (−33–−24 bp: AGCACATATA). Additionally, a chromatin immunoprecipitation (Ch-IP) assay involving N2A cells was conducted to confirm whether NPAS4 protein interacts with the Nlrp6 promotor sequence. PCR primers for site2 were designed to testify the Ch-IP products. As shown in Figure 4I, the amplified fragments were around 100–200 bp in length. In addition, the amplification of *Nlrp6* promotor (site2) fragments was significantly higher in the anti-NPAS4 group than the IgG group that act as the negative control (*p* < 0.0001), which illustrated that NPAS4 protein can interact with the Nlrp6 promotor sequence at site2.

Taken together, the NPAS4 transcription factor may promote inflammasome activation in the acute phase of ICH by interacting with the Nlrp6 promotor.

### 2.5. NPAS4 Overexpression Aggravated Neuron Death, Neurological Deficits, and Cell Pyroptosis following ICH

**Figure 5:**
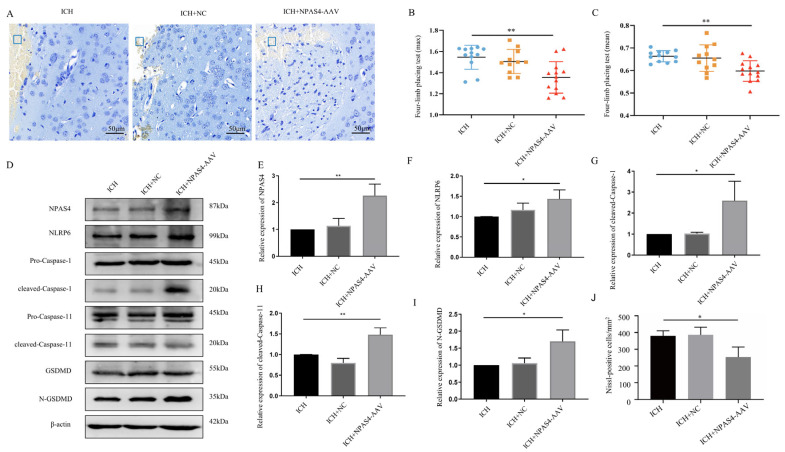
The effect of NPAS4 overexpression on neuron death, neurologic outcomes, and pyroptosis post-ICH. (**A**,**J**) Nissl staining was performed to evaluate neuronal injuries around hematoma (scale bar = 50 μm; frame, hematoma); (**B**,**C**) grip strength test was conducted to assess neurological function (ICH, *n* = 12; ICH + NC, *n* = 11; ICH + NPAS4-AAV, *n* = 13); (**D**–**I**) Western blotting was used to detect the protein levels of NPAS4, NLRP6, and pyroptosis-related molecules. ICH, mice without injection before ICH induction; ICH + NC, mice with mock-AAV (no vector) injection one month before ICH induction; ICH + NPAS4-AAV, mice with NPAS4-AAV injection one month before ICH induction. * *p* < 0.05 vs. ICH; ** *p* < 0.01 vs. ICH.

To verify the detrimental function of NPAS4 in ICH, we injected NPAS4-overexpressing AAV or mock-AAV (no vector) into the right basal ganglia of wild-type mice one month before ICH modeling. As Figure 5E displays, *Npas4*-AAV injection significantly increased NPAS4 expression after ICH (*p* < 0.01), and NPAS4 overexpression exacerbated a series of ICH-induced injuries. A forelimb-placing test (Figure 5B,C) revealed that the *Npas4*-AAV-injected group (*n* = 13; max values—1.35 ± 0.15 N; mean values—0.60 ± 0.05 N) had more severe muscle weakness than the ICH (*n* = 12; max values—1.55 ± 0.11 N; mean values—0.66 ± 0.02 N) and ICH + NC (*n* = 11; max values—1.51 ± 0.11 N; mean values—0.66 ± 0.06 N) groups. Nissl staining (Figure 5A,J) demonstrated that the number of Nissl-positive neurons were visibly decreased in the NPAS4 overexpression group compared with the other two groups. Western blotting (Figure 5D–I) revealed that the protein levels of NLRP6, cleaved-capase-1, cleaved-capase-11, and N-GSDMD were highly upregulated in the perihematomal tissue of the *Npas4*-AAV injected mice in contrast to the ICH control (*p* < 0.05). And the protein levels of GSDMD-FL, pro-Caspase-1 and pro-Caspase-11 were showed no obvious differences among the three groups (Supplementary Figure S3D–F; *p* > 0.05). The data above clearly uncovered that NPAS4 overexpression aggravated neurologic function deficit, brain injuries, and cell pyroptosis. In summary, all the results implied that NPAS4 tends to participate in the development of ICH through promoting pyroptosis induced by NLRP6 inflammasome activation.

## 3. Discussion

After ICH occurs, both primary and secondary brain injuries can contribute to cell death, which includes pyroptosis, necroptosis, ferroptosis, and autophagy [31,32,33]. As one of the major forms of pathological cell death, pyroptosis is characterized by cell membrane pore formation and rupture, nuclear DNA breakage, chromatin condensation, and so forth [34,35]. An increasing number of studies have shown that alleviating neuron pyroptosis plays a significantly neuroprotective role against brain damage, which suggests that pyroptosis inhibition may represent a potential therapeutic strategy for patients with ICH [36,37,38]. Research on periodontal disease and hepatocellular carcinoma has suggested that NLRP6 might be involved in pyroptosis [39,40]. Moreover, our previous study discovered that NLRP6 aggravated cell pyroptosis and neuronal damage in an OGD model [41]. In addition, we found that NLRP6 expression was upregulated and, subsequently, exaggerated brain injury after ICH in rats [23]. Moreover, we predicted that there are three theoretical NPAS4-binding sites in the *Nlrp6* promoter region using a bioinformatics prediction approach. Therefore, this study was designed to explore whether NPAS4 affects intracerebral hemorrhage and interacts with the *Nlrp6* promoter.

NPAS4 is a specific neuronal transcription factor that is highly expressed in the nervous system [42]. An increasing number of studies have shown that NPAS4 plays a critical role in nervous system development and diseases [30]. In this study, we found that NPAS4 protein levels were increased in human and mouse peri-hematoma brain tissues. Similarly, it has been reported that NPAS4 was upregulated in a series of brain damages such as focal and global ischemic stroke [43,44]. A previous study demonstrated that NPAS4 is important for long-term memory formation in two regions of the brain: the hippocampus and amygdala [45]. However, several recent studies have revealed the complex function of NPAS4. A significantly larger lesion size and more severe neurodegeneration in Npas4^−/−^ mice following cerebral ischemia modeling were reported, thus confirming the neuroprotective role of NPAS4 in ischemic stroke [46]. Alternatively, SST-specific Npas4-KO mice exhibited relatively minor anxiety-like behavior, which indicated that NPAS4 might also act as a harmful factor [47]. Consistently, our study showed that the knockdown of NPAS4 alleviated neutrophil infiltration, cell pyroptosis, brain damage, and neurological deficits within 24 h after ICH, while the overexpression of NPAS4 was obviously able to reverse these alternations, suggesting that NPAS4 might play an injurious role in the acute phase of ICH.

NLRP6 can recruit apoptosis-associated speck-like proteins containing a CARD (ASC) and promote the activation of Caspase-1 [48]. The pore-forming protein GSDMD, the executor of pyroptosis, can be cleaved and activated by Caspase-1 [49]. Moreover, a recent study demonstrated that NLRP6 can recruit caspase-1 and caspase-11 via the adaptor ASC in response to cytosolic Lipoteichoic acid (LTA), while caspase-11-mediated NLRP6 cleavage promoted caspase-1 activation in macrophagic bacteria [22]. In our present study, we found that the inhibition of NPAS4 downregulated the expression of NLRP6 and decreased the activation of Caspase-1, Caspase-11, and N-terminal GSDMD, while the overexpression of NPAS4 upregulated the expression of NLRP6 and increased the activation of Caspase-1, Caspase-11, and N-terminal GSDMD. Thus, NPAS4 may exacerbate cell pyroptosis via upregulating NLRP6 expression and then activating the inflammasome. In addtion, NPAS4’s N-terminal, which contains a bHLH domain, could dimerize with other BHLH-PAS proteins to form the DNA-binding interface of functional dimers and further regulate the expression of target genes (e.g., Bdnf, Narp, Kcna1) under different stress conditions, which mediate the diverse effects of Npas4 on synapses [50,51]. Notably, our study is the first to discover that NPAS4 can interact with the *Nlrp6* promoter region (−400–−391 bp and −33–−24 bp) and subsequently upregulate the mRNA expression of *Nlrp6*.

Remarkably, there are still some limitations to our current study. We investigated the effects of NPAS4 on mice subjected to experimental ICH and *Nlrp6* transcriptional levels, but did not intervene in NLRP6 expression simultaneously so as to further clarify the regulatory function of NPAS4 in an NLRP6-induced brain injury. In follow-up experiments, Nlrp6^−/−^ mice should be raised to further explore the interaction of NPAS4 with the NLRP6 inflammasome in order to determine the potential mechanism of NPAS4 after intracerebral hemorrhage. Moreover, NPAS4 plays a crucial role in neurogenesis and memory formation [52]. We focused on the role and mechanism of NPAS4 in the acute phase of ICH in mice, but we did not explore its further function during the chronic and recovery stages of ICH. It is worth mentioning that NPAS4 works in the form of homo- and heterodimers rather than activating alone and interacts with differential proteins involved in signal transduction pathways, performing a variety of extensive and complicated regulatory functions [53,54]. Furthermore, there is still some ambiguity regarding the specific molecular mechanism of activation and interaction between NPAS4 and other proteins. Thus, it is necessary to continuously research NPAS4 to better understand the development and progression of diseases and provide guidance for clinical treatments.

**Figure 6:**
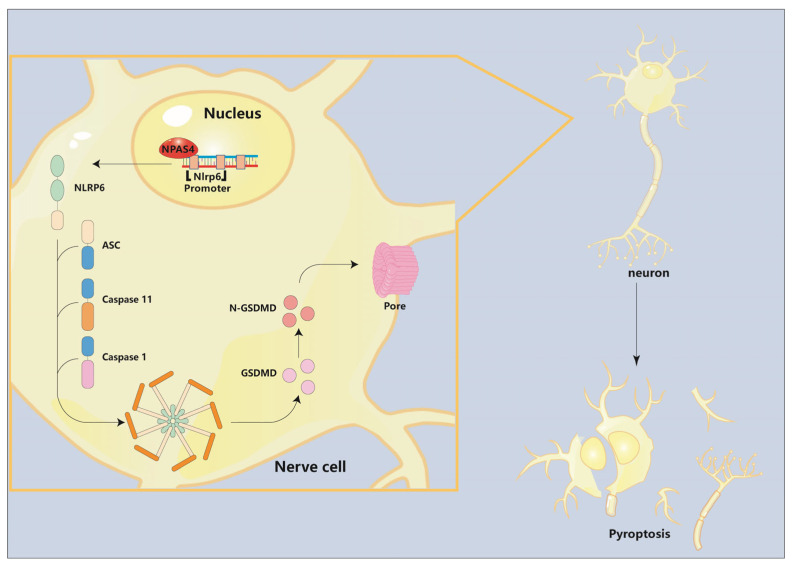
The role of Npas4 in pyroptosis after ICH. The pattern diagram shows that Npas4 promotes ICH-induced pyroptosis via transcriptionally activating Nlrp6.

In summary, in this study, we have demonstrated the role and underlying mechanism of NPAS4 in the acute phase of intracerebral hemorrhage in mice. Firstly, our data showed that NPAS4, which is highly expressed in neurons, was upregulated in human and mouse peri-hematoma brain tissues and peaked at approximately 24 h after ICH modeling. Secondly, we found that NPAS4 exacerbated neutrophil infiltration, cell pyroptosis, brain injury, and neurological dysfunction at 24 h post-ICH. Moreover, NPAS4 regulated the expression of NLRP6, cleaved Caspase-1, cleaved Caspase-11, and N-terminal GSDMD. Lastly, it was observed that NPAS4 could interact with the *Nlrp6* promoter −400–−391 bp and −33–−24 bp regions to activate *Nlrp6* transcription (Figure 6).

## 4. Materials and Methods

### 4.1. Human Tissues

This study was approved by the Ethics Committee of Children’s Hospital of Chongqing Medical University (File No. 2021295) and conducted in compliance with the ethical standards outlined in the Declaration of Helsinki. Brain tissues were obtained from spontaneous intracerebral hemorrhage patients (*n* = 3). Control tissues were provided by the Department of Forensic Medicine, Chongqing Medical University (*n* = 3). The details are shown in Supplementary Table S1.

### 4.2. Animals

Adult male C57BL/6 mice (6–8 w), which were provided by the Laboratory Animal Center of Chongqing Medical University (Chongqing, China), were maintained in a standardized environment with controlled temperature (25 °C) and humidity (relative humidity 55%) and provided ad libitum access to food and water under a 12 h light/dark cycle. All animal procedures and protocols were approved by the Institutional Animal Ethics Committee of Chongqing Medical University and were conducted in accordance with Institutional Animal Care and Use Committee (IACUC) guidelines (NIH Publication No. 85-23, revised 1996).

### 4.3. ICH Model

Modeling intracerebral hemorrhage in mice: firstly, mice were anesthetized with sodium pentobarbital via intraperitoneal injection and then positioned on a stereotaxic apparatus (RWD Life Science Co., Ltd., Shenzhen, China). After having their hair cut and disinfected by 75% ethanol, mice were made a small sagittal incision and their skulls were exposed extensively with hydrogen peroxide in order to drill a burr hole on the right side of the skull (anteroposterior (AP), −0.2 mm; mediolateral (ML), −2.0 mm). Second, autologous blood (30 µL) from the tail-tips of the mice obtained via a Hamilton micro-syringe rinsed with heparin was automatically infused into the right basal ganglia (dorsoventral (DV), −3.5 mm) at a rate of 1.5 µL/min with micro-infusion pump. Subsequently, micro-syringe was maintained in situ for an additional 10 min to minimize blood reflux and was then withdrawn slowly. Lastly, the burr hole was covered with bone wax, and the wounded scalp was sutured and sterilized again with 75% ethanol. Additionally, the sham group was injected with an equal volume of sterile saline solution.

### 4.4. siRNA Transfections

NPAS4 siRNA (si-503 (forward: 5′-CGUUUCUGAAAGUGUCCUAAUTT-3′; reverse: 5′-AUUAGGACACUUUCAGAAACGTT-3′); si-848 (forward: 5′-CCUGAUAAUUUAUUCCUGGAATT-3′; reverse: 5′-UUCCAGGAAUAAAUUAUCAGGTT-3′); si-2253 (forward: 5′-CGACAGUAGCUACGAUAUCAUTT-3′; reverse: 5′-AUGAUAUCGUAGAUACUGUCGTT-3′)) and scramble siRNA as a negative control were designed and synthesized by Sangon Biotech Company (Shanghai, China). NPAS4 siRNA (0.05 OD/µL) was pumped at a rate of 1.0 µL/min within 10 min into the right lateral ventricle (anteroposterior (AP), −0.3 mm; mediolateral (ML), −1.2 mm; dorsoventral (DV), −2.5 mm) and transfected 24 h before ICH modeling.

### 4.5. Adeno-Associated Virus Transfection

*Npas4* gene was packaged into the Vector (pcAAV-CMV-EGFP-P2A-*Npas4*-3xFLAG-WPRE) for overexpression (AAV-*Npas4*). A plasmid (pcAAV-CMV-EGFP-P2A-MCS-3xFLAG-WPRE) not encoding NPAS4 was used as a control AAV vehicle (AAV-NC). Both were purchased from Obio Technology Corporation (Shanghai, China). Adeno-associated viruses, namely, AAV-*Npas4* (1.30 × 10^12^ vg/mL, 2 µL) or AAV-NC (3.70 × 10^12^ vg/mL, 2 µL), were administered in the right basal ganglia (anteroposterior (AP), −0.2 mm; mediolateral (ML), −2.0 mm; dorsoventral (DV), −3.5 mm) over 10 min one month before the establishment of ICH model.

### 4.6. Grip Strength Test

To evaluate the neurological function of the murine models, a grip strength test was conducted using a grip strength meter (Sansbio, Nanjing, China) that recorded the maximum and average force. The forces were measured in Newtons. During the test, the grip strength meter was positioned horizontally, and the mice were held by the tail and lowered towards the apparatus. For the four-limb placing test, mice were allowed to grasp the metal grid with four limbs; for the forelimb placing test, mice were allowed to hold the metal grid with their forelimbs, while their hindlimbs were not allowed to make contact with the grid. Then, mice were gently pulled backwards by the tail until they could no longer hold the grid. Grip strength test was carried out in triplicate for each mouse.

### 4.7. Hematoxylin and Eosin (H&E) Staining

H&E staining was used to assess brain edema and neuronal injury after ICH. After sequential perfusion of normal saline and 4% paraformaldehyde from apex of the heart into the whole body, the brains were extracted and soaked in 4% paraformaldehyde for 24 h fixation and then dehydrated with gradient ethanol. After being transparentized in dimethylbenzene and immersed in paraffin wax, the brains were sliced into 5 µm thick, paraffin-embedded coronal sections. Following deparaffinization and benzene removal, the brain sections were stained with hematoxylin–eosin and, in a gradient, dehydrated again so that they could be observed under a microscope.

### 4.8. Nissl Staining

Nissl staining was performed to measure neuronal loss after ICH. The brain sections were collected through the above-mentioned approach. After being debenzolized and deparaffinized, the brain slices were soaked in a tar purple solution (Sigma-Aldrich, Saint Louis, MO, USA) for 5–15 min and then decolorized in gradient ethanol for 2 min each. Finally, the slices were transparentized, sealed, and observed under a microscope.

### 4.9. Immunofluorescence Staining

Brain sections were collected through the above-mentioned approach. After being deparaffinized in xylene and dehydrated with gradient ethanol, brain slices were submerged in antigen retrieval solution and then heated in a high-pressure cooker for 3 min for antigen retrieval. Slices were allowed to cool to room temperature before proceeding. Brain slices were washed three times with phosphate-buffered saline (PBS) and permeabilized with 0.4% Triton X-100 for 30 min. After being blocked with 5% goat serum (Boster Biological Technology, Wuhan, China) for 30 min at room temperature (RT), slices were incubated with primary antibodies overnight at 4 °C and, subsequently, corresponding secondary antibodies (Servicebio Technology, Wuhan, China) in the dark for 1 h at RT after being washed three times with PBS. Lastly, the slices were counterstained with 4′,6 diamidino-2-phenylindole (Solarbio Life Science, Beijing, China). TUNEL assay was conducted using a TUNEL Apoptosis Detection Kit (Servicebio Technology, Wuhan, China). The primary antibodies used included rabbit anti-Iba-1 (Abcam, Boston, MA, USA), rabbit anti-NeuN (Abcam, Boston, MA, USA), rabbit anti-GFAP (Abcam, Boston, MA, USA), goat anti-NPAS4 (Novus, Littleton, CO, USA), rabbit anti-MPO (Abcam, Boston, MA, USA), and rabbit anti-Caspas-1 (Abcam, Boston, MA, USA). Images were captured with a fluorescence microscope (Nikon, Tokyo, Japan) and analyzed using ImageJ software (1.48v).

### 4.10. Immunohistochemical Staining

Immunohistochemical staining was conducted according to the universal two-step test kit (PV-9000; ZSGB, Beijing, China). Briefly, 5 µm sections of the brain tissues were baked at 70 °C for 2 h. Then, the sections were de-paraffinized in xylene, rehydrated using a gradient of ethanol concentrations, boiled in citrate antigen retrieval solution in a high-pressure cooker for 3 min for antigen retrieval, blocked with 3% hydrogen peroxide for 15 min to inhibit endogenous peroxidase activity, and incubated with goat serum for 30 min to reduce background non-specific staining. Subsequently, sections were incubated with goat anti-NPAS4 (1:100; Novus, Littleton, CO, USA) overnight at 4 °C and then a corresponding secondary antibody at room temperature for 15 min. Subsequently, color development was determined using a DAB Substrate kit (ZLI-9018; ZSGB, Beijing, China). Lastly, the sections were counterstained with hematoxylin, dehydrated, transparentized, and sealed. The intensity of immunostaining was scored as 0 (no immunostaining), 1 (weak immunostaining), 2 (moderate immunostaining), or 3 (strong immunostaining). The percentage of positive cells was scored as 0 (<5%), 1 (5–25%), 2 (26–50%), 3 (51–75%), or 4 (76–100%). Finally, the IHC score for each sample was calculated as the intensity of the immunostaining score multiplied by the percentage-of-positive-cells score.

### 4.11. Western Blotting

Brain tissue around hematoma at different timepoints after ICH was collected for protein homogenate extraction. Protein concentration was quantified using the BCA method with a commercial kit (Beyotime, Shanghai, China). A total of 30 µg of protein from each group was electrophoresed on 10% SDS-PAGE gel and transferred onto polyvinylidene difluoride (PVDF) membrane. The membranes were incubated at 4 °C overnight with primary antibodies, which was followed by incubation with corresponding anti-goat (1:2000; Novus, Littleton, CO, USA) or anti-rabbit (1:5000; Abways, Shanghai, China) secondary antibodies for 2 h at room temperature. Enhanced chemiluminescence reagent kits (Affinity, Waltham, MA, USA) were used to visualize the bands. Integrated optical density (IOD) values were measured for the quantification of target protein using ImageJ software (1.48v) and expressed as a relative value normalized to β-actin levels. The following primary antibodies were used: goat anti-NPAS4 (1:700; Novus, Littleton, CO, USA), rabbit anti-β-actin (1:5000; Abways, Shanghai, China), rabbit anti-IL-6 (1:200; Santa Cruz, Dallas, TX, USA), rabbit anti-TNF-α (1:200; Santa Cruz, Dallas, TX, USA), rabbit anti-IL-1β (1:1000; Abclonal, Wuhan, China), rabbit anti-N-GSDMD (1:1000; Abclonal, Wuhan, China), rabbit anti-GSDMD (1:1000; Abclonal, Wuhan, China), rabbit anti-cleaved-Caspase-1(1:500; Cell Signaling Technology, Boston, MA, USA), rabbit anti-pro-Caspase-11 (1:1000; Abclonal, Wuhan, China), and rabbit anti-NLRP6 (1:1500; Abclonal, Wuhan, China).

### 4.12. Quantitative Real-Time PCR (qRT-PCR)

Total mRNA was extracted from isolated brain tissues using trizol reagent. qRT-PCR was performed in duplicate using the SYBR Green method, and the transcriptional level of glyceraldehyde 3-phosphate dehydrogenase (GAPDH) was used as an internal control. mRNA expression levels of candidate genes were calculated using 2^−ΔΔCT^ method. Primers used for the analysis are listed as follows: NLRP6—sense (CGGGACGAGAGGAAGGAGAG), antisense (CACACGATCCAGCACACGAGG); NLRP6(ChIP-PCR)—sense (GCTTGGCTTTGTATTTCTTCCCTT), antisense (TGGTTTCCTGATCCCACATGAAG); GAPDH—sense (GATGCAGGGATGATGTTCTG), antisense (GTGAAGGTCGGTAACGG).

### 4.13. Plasmids Construction

DH5α *E. coli* strain was used for plasmid construction. Liquid medium used for bacterial cultures in this study was Luria–Bertani–Miller (LB) broth. Premixed antibiotic solution of penicillin and streptomycin was purchased from Gibco (Waltham, MA, USA). T4 DNA ligase was acquired from Takara Biomedical Technology (Beijing, China). Phusion High-Fidelity DNA Polymerase and all restriction enzymes were purchased from New England BioLabs (Ipswich, MA, USA). Genomic DNA templates of a murine origin were obtained from Qi, H. at Basic Medical College, Chongqing Medical University. All plasmids were constructed using basic molecular cloning methods, including standard steps such as PCR, restriction digestion, ligation, and transformation. In brief, to generate NPAS4 overexpression plasmids (H340-*Npas4*-Flag), oligonucleotide primers (Supplementary Table S2) with NheI and XhoI restriction enzyme sites and corresponding protective base pairs were designed using SnapGene4.3.6 and then synthesized by Tsingke Biotechnology Company (Beijing, China). After being amplified via PCR and then electrophoresed in 2% agarose gel, desired target fragments were identified and recovered using an agarose gel extraction kit (Promega, Madison, WI, USA). Then, the amplified DNA products and the plasmid H340 were digested simultaneously by NheI and XhoI, followed by ligation with T4 DNA ligase kit (Takara, Dalian, China). For transformation, we used standard heat shock applied to E. coli DH5α and then sent selected independent colonies for sequencing. After identification, plasmids were purified with plasmid extraction kit (Macherey-Nagel, Düren, Germany). For plasmid construction of pGL4.10-*Nlrp6* promotor(fFluc) and pGL4.10-*Nlrp6* promotor mutant (fFluc), first, *Nlrp6* gene promotor regions (segment 1: −1902–+496, segment 2: −987–+496, and segment 3: −210–+496) were obtained from the NCBI online database and then corresponding NPAS4 binding sites (site 1: −1342–−1333 bp, site 2: −400–−391 bp, site 3: −33–−24 bp) were predicted using the Jaspar2020 website. Particularly, mutations in the predicted binding sites were performed using base mutation from A/T to G/C and vice versa, and then the primers (see Supplementary Table S2) were designed with NheI and KpnI restriction sites and protective base pairs. The next steps were performed as described above.

### 4.14. Dual Luciferase Reporter Gene Assay

N2A cells were co-transfected with the pcDNA pIRES2-NPAS4-Flag plasmid, which overexpressed NPAS4 or pcDNA pIRES2-Flag as a control vector, together with pGL4.10-Nlrp6 promotor(fLuc) and pGL4.74(rLuc) plasmids. After transfection, cells were harvested, and lysates were used to detect firefly and renilla luciferase activity using dual luciferase reporter gene assay kits (Hanbio, Shanghai, China) (used according to the manufacturer’s protocol). Normalized values (firefly/renilla activity) were used for analysis.

### 4.15. Chromatin Immunoprecipitation (ChIP) Assay

ChIP assay was performed by employing the SimpleChIP^®^ Enzymatic Chromatin IP Kit (CST#9002, Boston, MA, USA), which was used according to the manufacturer’s instructions. In brief, N2A cells were fixed with 1% formaldehyde for 15 min at room temperature; then, MNase digestion was carried out. Cell lysate was sonicated for 5 min at 0 °C. Subsequently, protein lysate was incubated with anti-NPAS4 (Novus, Littleton, CO, USA), anti-histone H3, or normal IgG for immunoprecipitation overnight at 4 °C. After releasing crosslink, purified DNA was subjected to PCR (using the primers 5′-GCTTGGCTTTGTATTTCTTCCCTT-3′ and 5′-TGGTTTCCTGATCCCACATGAAG-3′) to amplify the −400 to −391 region of the mouse Nlrp6 promoter (site 2).

### 4.16. Statistical Analysis

GraphPad Prism software (version 8.0.2) was used for statistical analysis. Student’s *t* test was used between two groups. Normal one-way ANOVA followed by Tukey’s test were conducted for multiple comparisons. The data are presented as means ± SD. A *p* value < 0.05 was considered significant.

## 5. Conclusions

In conclusion, our study demonstrated for the first time that NPAS4 can interact with the Nlrp6 promoter region (−400–−391 bp and −33–−24 bp) and subsequently upregulate Nlrp6 transcription levels. Additionally, it also confirmed that NPAS4 might aggravate pyroptosis and brain injury via activating the NLRP6 inflammasome after intracerebral hemorrhage in mice.

## Data Availability

Not applicable.

## Supplementary Materials

The following supporting information can be downloaded at: https://www.mdpi.com/article/10.3390/ijms24098320/s1.
