# Supplementary material for: NPAS4 Exacerbates Pyroptosis via Transcriptionally Regulating NLRP6 in the Acute Phase of Intracerebral Hemorrhage in Mice
Source: Int J Mol Sci. 2023 May 5;24(9):8320. doi: 10.3390/ijms24098320 (PMC10179070; doi:10.3390/ijms24098320)
Supplement: Supplementary file 1 [file ijms-24-08320-s001.zip › ijms-2337392-supplementary.pdf]

Supplementary Materials

Supplementary Table S1. Human tissue information.

Sample	sex	age	Possible bleeding causes	ICH location	Sample collection time	Estimated ICH volume
ICH 1	M	11	Arteriovenous malformation	Left parietal-occipital lobe	20 hours after symptom onset	40mL
ICH 2	F	11	Cerebrovascular malformation	Left frontotemporal lobe	25 hours after symptom onset	30mL
ICH 3	F	10	Arteriovenous malformation	Midline area, left parietal lobe	19 hours after symptom onset	30mL
CTR1	M	New-born	-	-	-	-
CTR2	M	59	-	-	-	-
CTR3	F	60	-	-	-	-

Supplementary Table S2. Primer sequences of plasmids construction.

Gene name	Species	Primer sequences
NPAS4 overexpression plasmids(H18894)		
NPAS4	*Mus*	Forward: CTAGCTAGCATGTACCGATCCACCAAGGG
		Reverse: CCGCTCGAGAAACGTTGGTTCCCCTCCAC
pGL4.10-Nlrp6 promotor		
NLRP6 (segment 1)	*Mus*	Forward: GGGGTACCTGGTCATATTTGTATGTCTGTGCC
		Reverse: CTAGCTAGCTAGCTTCCCTACGTGGGTCT
NLRP6 (segment 2)	*Mus*	Forward: GGGGTACCGCTTGGCTTTGTATTTCTTC
		Reverse: CTAGCTAGCTAGCTTCCCTACGTGGGTCT
NLRP6 (segment 3)	*Mus*	Forward: GGGGTACCGCCCTCTTGATTTTCACGCC
		Reverse: CTAGCTAGCTAGCTTCCCTACGTGGGTCT
pGL4.10-Nlrp6 promotor mutant		
NLRP6 (site 1)	*Mus*	Forward: CCTTATACTTCACTAACATGCCGATGAATACCATAAGCG
		Reverse: CGCTTATGGTATTCATCGGCATGTTAGTGAAGTATAAGG
NLRP6 (site 2)	*Mus*	Forward: GTGGAGGTGTGACACATGTAATTTACACACGTC
		Reverse: ACGGACGTGTGTAAATTACATGTGTCACACCTCCA
NLRP6 (site 3)	*Mus*	Forward: AAAGGTCGCAGTCCCTACACGCGCTGATGTTACAA
		Reverse: TTGTAACATCAGCGCGTGTAGGGACTGCGACCTTT
Ligation segments	*Mus*	Forward: GGGGTACCTGGTCATATTTGTA
		Reverse: CTAGCTAGCTAGCTTCCCTACGT

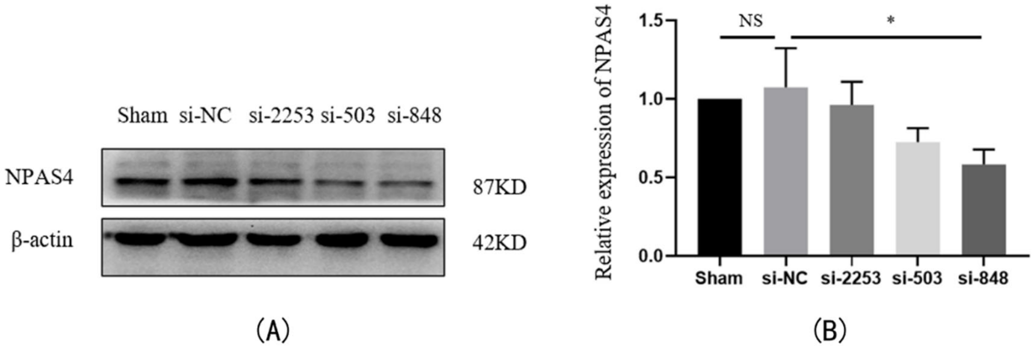

Supplementary Figure S1. Western blot was used to screen out the optimum interference fragments for NPAS4 in C57BL/6 and relative protein level was normalized to β-actin. *p<0.05 vs si-NC. As the figures showed, group of si-848 injection represented the most interference effect on NPAS4 expression level among four groups.

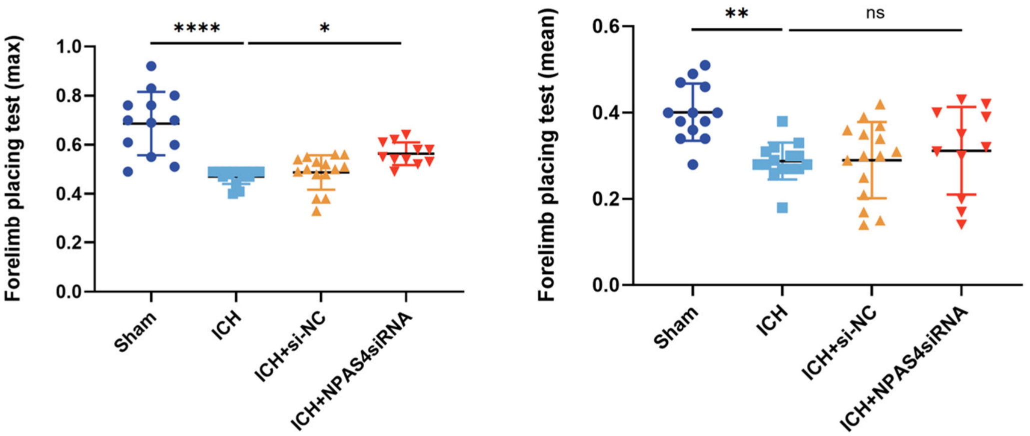

Supplementary Figure S2. Grip strength test (forelimb placing test) was conducted to assess neurological function (max and mean value were calculated). Sham, mice without any treatments, n=13; ICH, mice without injection before ICH induction, n=15; ICH+NC, mice with scrambled siRNA injection at 24h before ICH induction, n=15; ICH+NPAS4-siRNA, mice with NPAS4-siRNA injection at 24h before ICH induction, n=11. The data are shown as mean ± SD. *p<0.05 vs. ICH, **p < 0.01 vs. Sham, ****p < 0.0001 vs. Sham. As the pictures above displayed, forelimb muscle strength was significantly decreased in ICH group (max values,0.47±0.03N; mean values,0.29±0.04N) compared with sham group (max values,0.67±0.13N; mean values,0.40±0.07N), which was consistent with the data of four-limb strength test we've done before. Similarly, ICH+NPAS4-siRNA group (max values,0.56±0.05N) showed increased max forelimb strength in comparison with ICH group. On the contrary, mean forelimb strength was not improved when compared ICH+NPAS4-siRNA group (mean values,0.31±0.10N) to ICH group. Anyway, the difference was probably caused by inappropriate experimental manipulations or uncooperative mice.

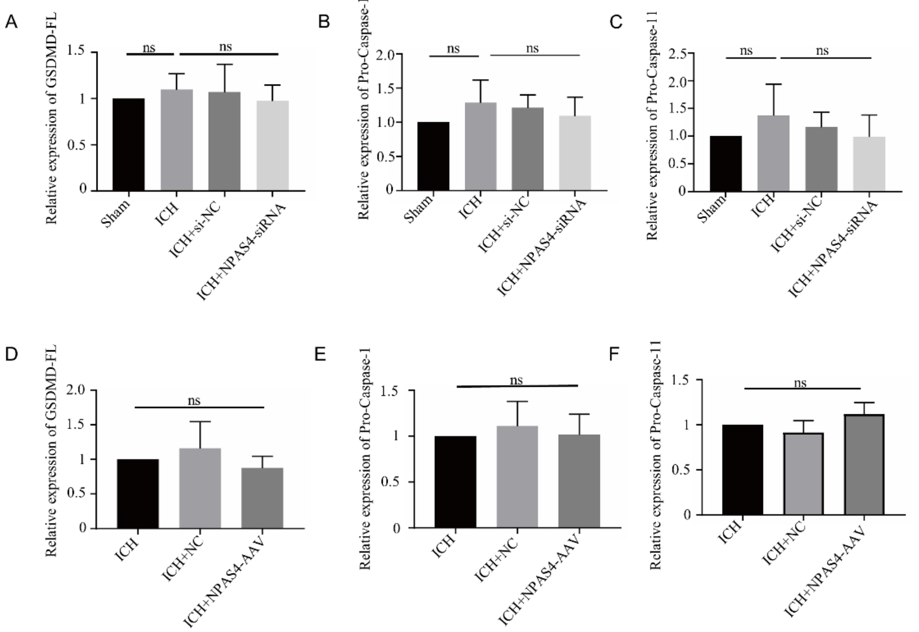

Supplementary Figure S3. Effect of regulating NPAS4 on the protein levels of GSDMD-FL, Pro-Caspase-1 and Pro-Caspase-11 at 24h after ICH. (A-C) Western blot was used to detect the protein levels of GSDMD-FL, Pro-Caspase-1 and Pro-Caspase-11 after NPAS4 inhibiting, n=4/group; (D-F) Western blot was used to detect the protein levels of GSDMD-FL, Pro-Caspase-1 and Pro-Caspase-11 after NPAS4 overexpression, n=3/group. The relative protein level was normalized to β-actin. ns, $p>0.05$. As the figures showed, the densitometry results of Pro-caspase-1, Pro-caspase-11, and GSDMD-FL did not show significant differences among the treatment groups.
